# Preliminary results of Preserflo Microshunt versus Preserflo Microshunt and Ologen implantation

**DOI:** 10.1186/s40662-021-00253-3

**Published:** 2021-09-03

**Authors:** Iraklis Vastardis, Sofia Fili, Georgios Perdikakis, Kalliopi Kontopoulou, Miltos Balidis, Zisis Gatzioufas, Markus Kohlhaas

**Affiliations:** 1 Department of Ophthalmology, St. Johannes Academic Hospital Dortmund, Dortmund, Germany; 2 Ophthalmica Eye Institute, Thessaloniki, Greece; 3 Department of Ophthalmology, University Hospital Basel, Basel, Switzerland

**Keywords:** Micro invasive glaucoma surgery, Ab externo surgery, Refractory POAG, SIBS polymer, Mitomycin C

## Abstract

**Purpose:**

To report preliminary 6-month results on the use of the Preserflo Microshunt implant with and without Ologen in 50 pseudophakic eyes with moderate to advanced primary open-angle glaucoma (POAG).

**Methods:**

Fifty pseudophakic eyes underwent ab externo Preserflo Microshunt implantation. Data was gathered retrospectively and two groups were then created, group A with application of MMC 0.2 mg/ml and group B with MMC 0.2 mg/ml and Ologen collagen matrix (OCM) implantation. Absolute success was regarded as the percentage of eyes achieving: a) 5 ≤ intraocular pressure (IOP) ≤ 13 mmHg, b) 5 ≤ IOP ≤ 16 mmHg, and c) 5 ≤ IOP ≤ 21 mmHg without additional medication or surgery and qualified success was regarded as the percentage of eyes achieving a) IOP ≤ 13 mmHg, b) IOP ≤ 16 mmHg, and c) IOP ≤ 21 mmHg with or without medication. Evaluation was performed using a log-rank Kaplan-Meier test. A scatterplot analysis presented the treatment effect data of all eyes with a minimum of 20% IOP reduction per case. Failure was defined as requiring additional surgery, IOP greater than 21 mmHg with or without medication and failure to reach 20% IOP reduction.

**Results:**

Mean postoperative IOP was significantly lower in both groups. IOP decreased by 49.06% in group A and by 53.01% in group B at 6 months (*P* < 0.88), respectively. Medication use was lower in both groups (Wilcoxon test, *P* < 0.001). The absolute and qualified success rates were not statistically significant between the groups (all *P* > 0.05). Cumulative IOP results per case were not statistically different in group A compared with group B. One revision surgery in group A (4% failure rate) and three in group B (12% failure rate) were performed.

**Conclusions:**

Both groups showed equal results in terms of cumulative and mean IOP reduction, medication reduction as well as in absolute and qualified success rates. No significant difference was found in any parameters tested between Preserflo Microshunt with MMC 0.2 mg/ml and with or without OCM implantation at 6 months. Long-term follow-up is required to further evaluate this data.

## Introduction

Glaucoma is still responsible for irreversible blindness globally [1]. Patient numbers around the world are rising and projected to reach 111.8 million soon [2]. Visual field deterioration in such patients can be prevented by lowering the intraocular pressure (IOP) which is achieved either through medication, laser treatment or surgery [3–5]. Trabeculectomy is widely performed and still regarded as the gold standard treatment. Although the method itself has been rigorously improved over the years [6], its use among surgeons is declining in favor of newer micro invasive glaucoma surgery (MIGS) devices [7]. Surgeons appear to be favoring such alternatives due to their superior safety and efficacy profiles [8]. These glaucoma implants have been introduced as a minimally invasive surgical option to prevent further glaucoma deterioration. These devices provide an outflow pathway of the aqueous humor from the anterior chamber of the eye into the posterior subtenonal space, and thus reduce IOP by mimicking the mechanism of trabeculectomy, while simultaneously conferring a standardized IOP outcome and an enhanced safety profile [9–11]. Such a device is the Preserflo™Microshunt (Santen, Osaka, Japan) implant formerly known as the InnFocus Microshunt (Santen, Osaka, Japan). It is a minimally invasive glaucoma drainage device that forms a posterior filtration bleb under the conjunctiva and Tenon’s capsule. Preserflo Microshunt consists of a polymer, the poly-styrene-block-isobutylene-block-styrene (SIBS), a stretchy material, that provokes minimal inflammation and encapsulation in comparison to other materials used in glaucoma surgery such as silicone rubber and polypropylene [12, 13]. SIBS technology was introduced in 1999 through TAXUS® (Boston Scientific’s, Natick, Massachusetts, USA) as a coronary stent in surgical treatment and prevention of arterial restenosis [14]. Today, as a glaucoma micro-implant and through its unique thermo-formable features it confers multiple advantages over traditional thermoset materials such as silicone rubber [12, 13]. There are multiple clinical trials evaluating its efficacy which are currently underway in Europe, USA, Singapore, Japan and the Dominican Republic. This case series study aims to not only report preliminary 6-month results on the use of the Preserflo Microshunt in terms of mean IOP and medication reduction, but also to compare two different surgical approaches: implantation of the Preserflo Microshunt with mitomycin C (MMC, 0.2 mg/ml) treatment and implantation of the Preserflo Microshunt with MMC 0.2 mg/ml treatment and additional Ologen collagen matrix (OCM) implantation. OCM is a biodegradable porous collagen-glycosaminoglycan copolymer matrix implant claiming wound modulation among connective and epithelial tissues. It also serves as a spacer or barrier between the sclera surface and conjunctiva [15]. Although its true benefit in glaucoma surgery is still being debated [16, 17], OCM is currently being widely utilized extending beyond glaucoma surgery [18] to other ocular pathologies, for instance in pterygium excision where it modulates the wound healing of the conjunctiva and serves as an inhibitor of recurrence [19]. In this cohort, both the late wound healing modulation of the bleb and the benefit of space creation between the Preserflo Microshunt and the Tenon’s capsule were evaluated. Our null hypothesis was that the IOP should be better in the eyes that underwent additional OCM implantation in comparison with the eyes that did not, by reducing the fibrotic reaction in the bleb area. The alternative hypothesis was that the IOP was equally lowered through the Preserflo Microshunt implantation with MMC 0.2 mg/ml regardless of the additional OCM implantation. If the OCM implantation indeed reduces the fibrotic reaction in the bleb area either through its properties or by acting as a barrier or spacer, then a reduction of revision surgery, needling interventions and additional use of antimetabolites or chemotherapeutical agents such as 5-fluorouracil (5-FU) should be reported.

## Material and methods

Fifty Caucasian patients and fifty eyes with treatment refractory primary open-angle glaucoma (POAG) were operated in 2020. Twenty-five eyes (group A) underwent a Preserflo Microshunt implantation with MMC (0.2 mg/ml), while the remaining 25 eyes (group B) underwent the same surgery with additional OCM implantation (Aeon Astron Europe BV, Leiden, The Netherlands). All surgeries were carried out under general anesthesia, performed by five Preserflo Microshunt certified surgeons, and took place in Saint Johannes Hospital, Ophthalmology Department, in Dortmund, Germany. All patients provided their written consent prior to surgery and the tenets of the Declaration of Helsinki were fully adhered to. The institution’s ethical committee approved this retrospective case series study. The primary patient selection criterion for this study was POAG refractory to medical treatment with prior uncomplicated cataract extraction. Inclusion and exclusion criteria are listed in Table 1. The baseline examination included a complete ophthalmic history, Humphrey visual field perimetry SITA Fast, endothelial microscopy (Tomey EM 4000, Tomey GmBH Technology and Vision, Nürnberg, Germany), Scheimpflug corneal tomography (Pentacam, OCULUS Optikgeräte GmbH, Germany), high-definition ocular coherence tomography (HD-OCT) optic disc and ganglion cell layer evaluation (Cirrus HD-OCT 500,Carl Zeiss Meditec AG, Germany), single measurement of calibrated Goldman applanation tonometry, Snellen visual acuity, slit-lamp anterior and posterior segment examination and gonioscopy with angle grading. Data was gathered again concurrently at the 1st day, 2nd week, 1st, 3rd, and 6th month including standard slit-lamp examination of the anterior and posterior segment, Goldman applanation tonometry, visual acuity testing and postoperative complications.

**Table 1:** Inclusion and exclusion criteria of the study cohort

**Inclusion Criteria**	
Refractory to medical treatment POAG and moderate to advanced POAG according to HPA classification system	
Prior uncomplicated cataract extraction, no prior history of glaucoma surgery or laser treatment	
ACD ≥ 2.7 mm, ACA ≥ Schaffer 3–4°	
**Exclusion Criteria**	
Secondary glaucoma, ACG, OHT, PDG, PEXG, LTG, prior glaucoma surgery or laser treatment	
Prior refractive surgery, PPV, phakic patients, corneal and retinal pathology, AMD, CME, last eye - monocular	
No glaucoma medication or mono-therapy	

*POAG* primary open-angle glaucoma; *HPA* Hodapp-Parrish-Anderson classification system; *ACD* anterior chamber depth; *ACA* anterior chamber angle; *ACG* angle-closure glaucoma; *OHT* ocular hypertension; *PDG* pigmentary dispersion glaucoma; *PEXG* pseudoexfoliation glaucoma; *LTG* low-tension glaucoma; *PPV* pars plana vitrectomy; *AMD* age-related macular degeneration; *CME* cystoid macular edema

### Surgical technique

For both groups, a 4-mm fornix based conjunctival peritomy was performed in the upper temporal or nasal quadrant in order to reveal the underlying sclera. Cauterization was performed under balanced salt solution (BSS) irrigation followed by scleral treatment with three MMC (0.2 mg/ml) soaked sponges posteriorly for three minutes. An ink marking was then placed 3 mm posterior to limbus followed by creation of an initial pocket with a 1.2-mm diamond blade. A 25-Gauge needle was then bent with the bevel up and inserted at a 90° angle into the anterior chamber, exiting at the level of the trabecular meshwork. The device was then slid through the pocket and tunnel while the fins of the device were stabilized into the scleral pocket. The aqueous humor flow chamber was tested with a sponge via observation. Finally, the device was covered from the conjunctiva and Tenon’s capsule, which in turn they were separately reattached with a 10–0 nylon running suture.

For group B exclusively, the 6 mm wide and 2 mm high OCM, was additionally placed posterior to the device’s fin and then tucked beneath the Tenon’s capsule and conjunctiva, prior to their reattachment with the 10–0 nylon running suture. Postoperative blebs were evaluated according to the Wuerzburg bleb classification score (WBCS) [20, 21]. It is based on a score that evaluates vascularization, corkscrew vessel and encapsulation but not the height of the bleb [22]. The postoperative medication in both groups was moxifloxacin three times daily and cycloplegics two times daily for the first week combined with preservative free corticosteroid eye drops instilled six times daily. After the first week, the antibiotics and cycloplegics were discontinued and the corticosteroid regimen was tapered by reducing one drop every week for the next 6 weeks.

### Statistical analysis

The mean IOP and medication reduction as well as the corrected distance visual acuity (CDVA) were evaluated with a two-way analysis of variance (two-way ANOVA) and paired samples t-test. Medication reduction between baseline and 6 months was reported using Wilcoxon test. Homoscedasticity, assumption of homogeneity, outliers assumption and the distribution of data for two-way ANOVA were also tested. Efficacy was assessed with the Kaplan-Meier survival analysis in three different postoperative IOP goals between the groups and with two different success criteria. Absolute success was regarded as the percentage of eyes that achieved a) 5 ≤ IOP ≤ 13 mmHg, b) 5 ≤ IOP ≤ 16 mmHg, and c) 5 ≤ IOP ≤ 21 mmHg without additional medication or surgery and qualified success was regarded as the percentage of eyes that achieved a) IOP ≤ 13 mmHg, b) IOP ≤ 16 mmHg, and c) IOP ≤ 21 mmHg with or without medication. We included a scatterplot analysis to present the treatment effect data of all eyes in this study that demonstrates the minimum 20% IOP reduction requirement achieved in both groups. Failure was defined as additional surgery, IOP greater than 21 mmHg with or without medication and failure to reach 20% IOP reduction. Cumulative IOP results per group were also evaluated and presented in percentages for IOP readings less or equal than 11, 13 and 15 mmHg. Intraoperative and postoperative complications as well as additional surgery rates were also evaluated as percentages. Statistical analysis was performed with MedCalc® 16.2.1 and IBM SPSS® version 22. Parametric or non-parametric tests were used according to distribution normality (Kolmogorov-Smirnov). We considered statistical significance as a *P* value < 0.05.

## Results

**Fig. 1:**
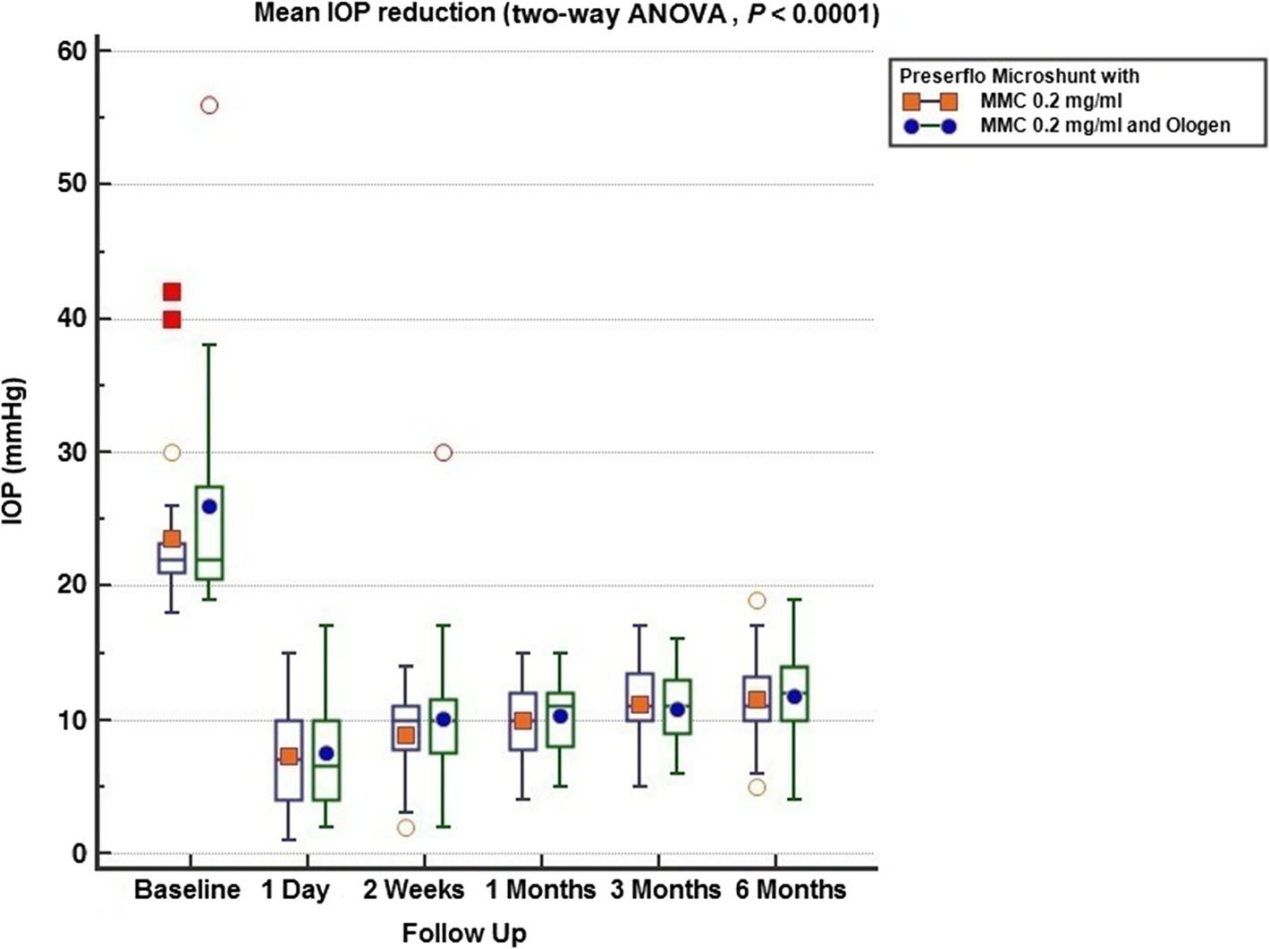
Box and Whisker plot of the mean intraocular pressure (IOP) reduction between Preserflo Microshunt with MMC 0.2 mg/ml and Preserflo Microshunt with MMC 0.2 mg/ml and Ologen implantation (two-way ANOVA, *P* < 0.001)

**Fig. 2:**
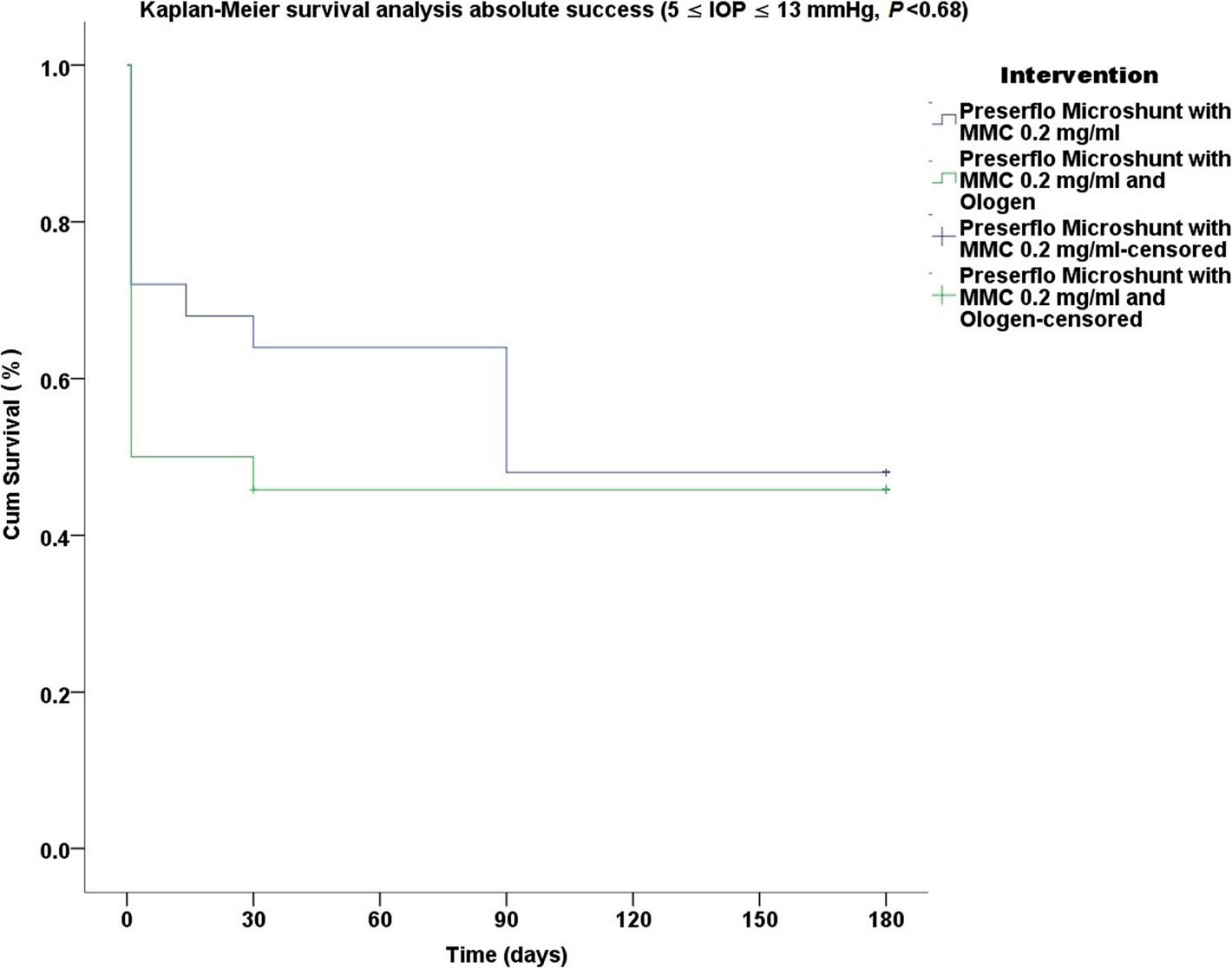
Log-rank, Kaplan-Meier survival analysis of absolute success rate between Preserflo Microshunt with MMC 0.2 mg/ml and Preserflo Microshunt with MMC 0.2 mg/ml and Ologen implantation (5 ≤ IOP ≤ 13 mmHg, *P* < 0.68)

**Fig. 3:**
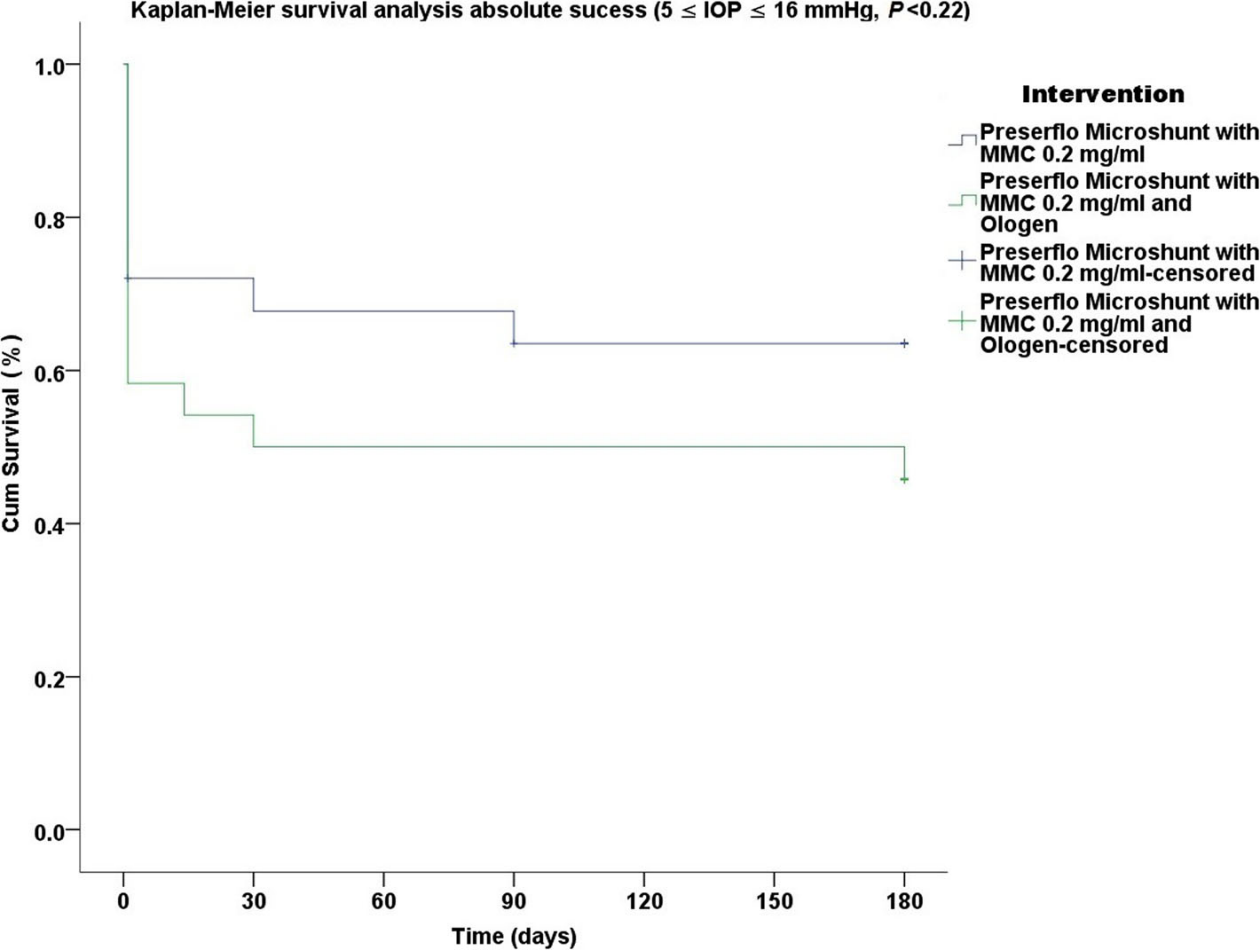
Log-rank, Kaplan-Meier survival analysis of absolute success rate between Preserflo Microshunt with MMC 0.2 mg/ml and Preserflo Microshunt with MMC 0.2 mg/ml and Ologen implantation (5 ≤ IOP ≤ 16 mmHg, *P* < 0.22)

**Fig. 4:**
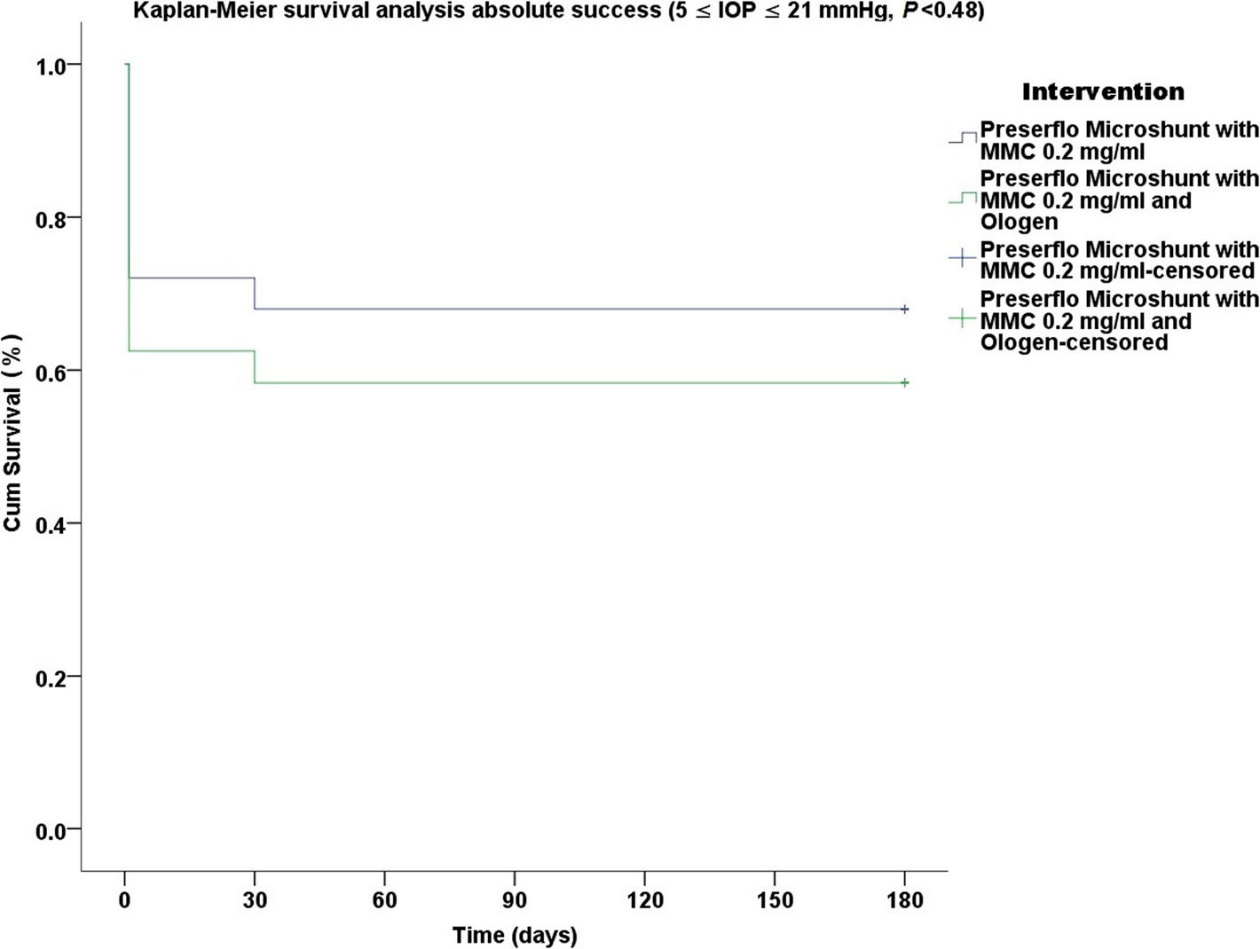
Log-rank, Kaplan-Meier survival analysis of absolute success rate between Preserflo Microshunt with MMC 0.2 mg/ml and Preserflo Microshunt with MMC 0.2 mg/ml and Ologen implantation (5 ≤ IOP ≤ 21 mmHg, *P* < 0.48)

**Fig. 5:**
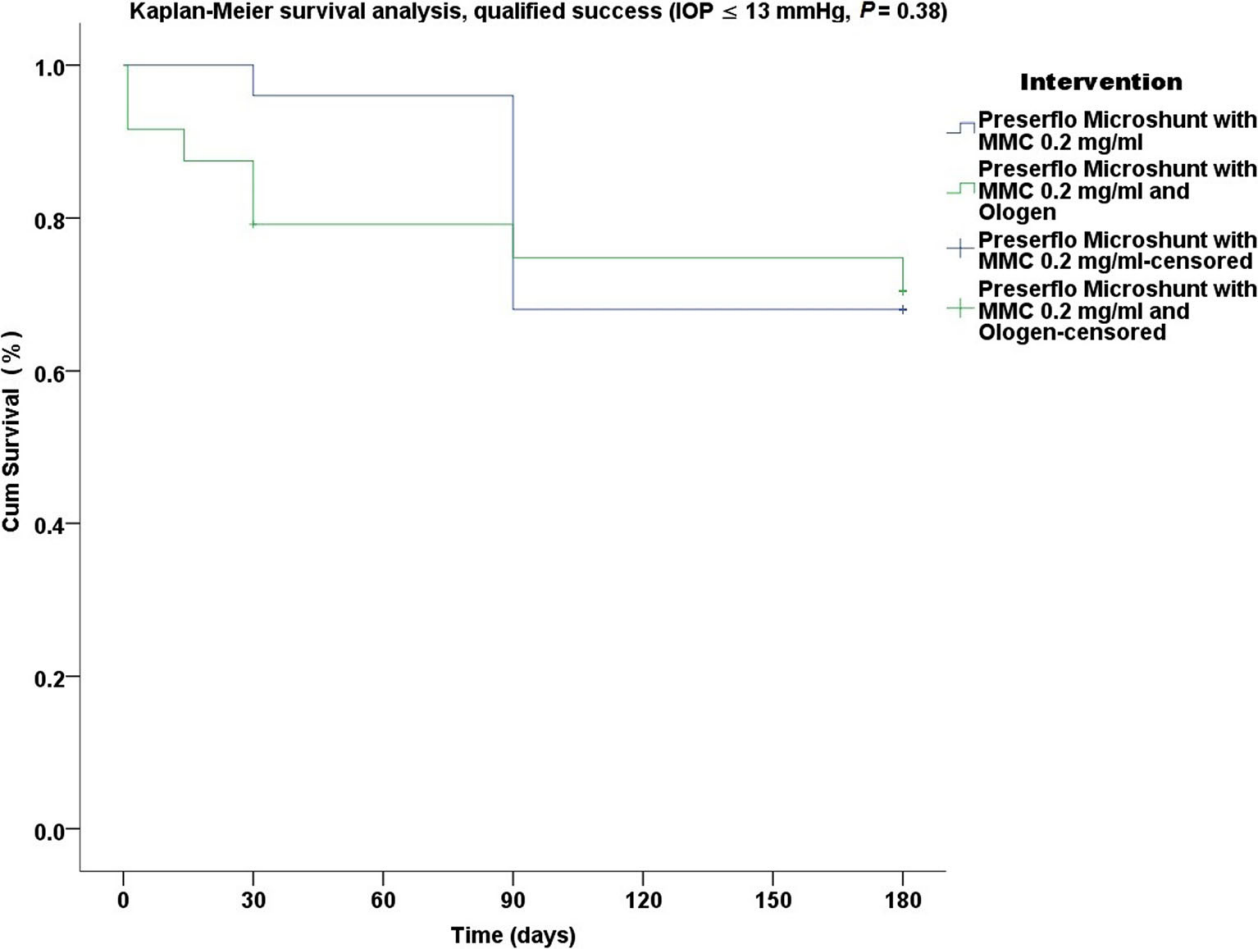
Log-rank, Kaplan-Meier survival analysis of qualified success rate between Preserflo Microshunt with MMC 0.2 mg/ml and Preserflo Microshunt with MMC 0.2 mg/ml and Ologen implantation (IOP ≤ 13 mmHg with or without medication, *P* = 0.38)

**Fig. 6:**
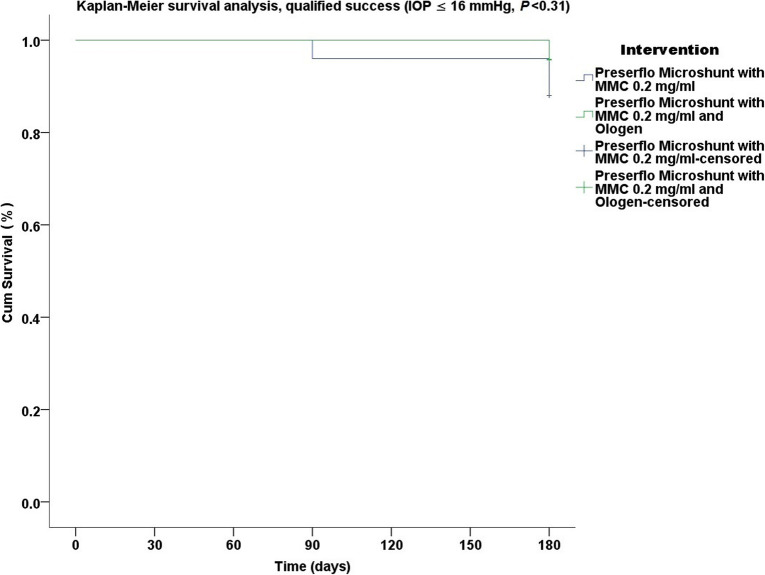
Log-rank, Kaplan-Meier survival analysis of qualified success rate between Preserflo Microshunt with MMC 0.2 mg/ml and Preserflo Microshunt with MMC 0.2 mg/ml and Ologen implantation (IOP ≤ 16 mmHg with or without medication, *P* < 0.31)

**Fig. 7:**
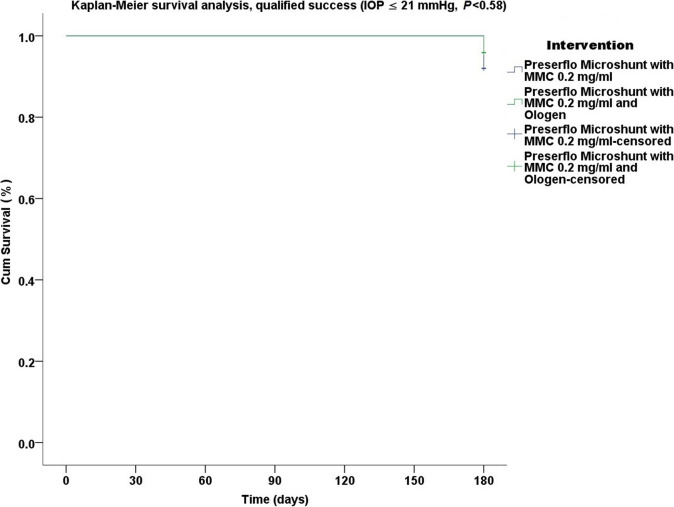
Log-rank, Kaplan-Meier survival analysis of qualified success rate between Preserflo Microshunt with MMC 0.2 mg/ml and Preserflo Microshunt with MMC 0.2 mg/ml and Ologen implantation (IOP ≤ 21 mmHg with or without medication, *P* < 0.58).

**Fig. 8:**
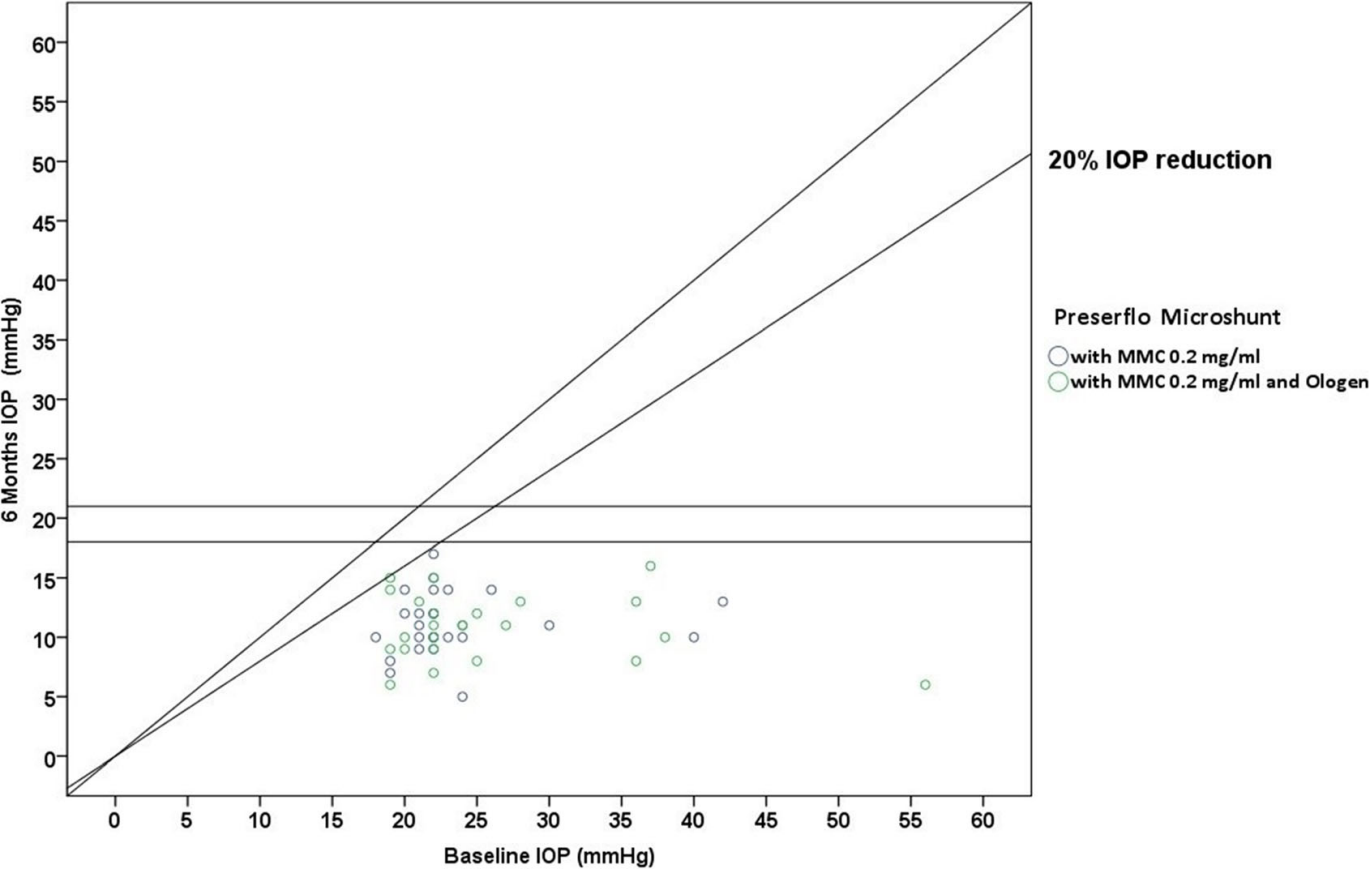
Scatterplot analysis of pressure control of effect data of all eyes (*n* = 50) between baseline and 6 months intraocular pressure (IOP) values.

**Fig. 9:**
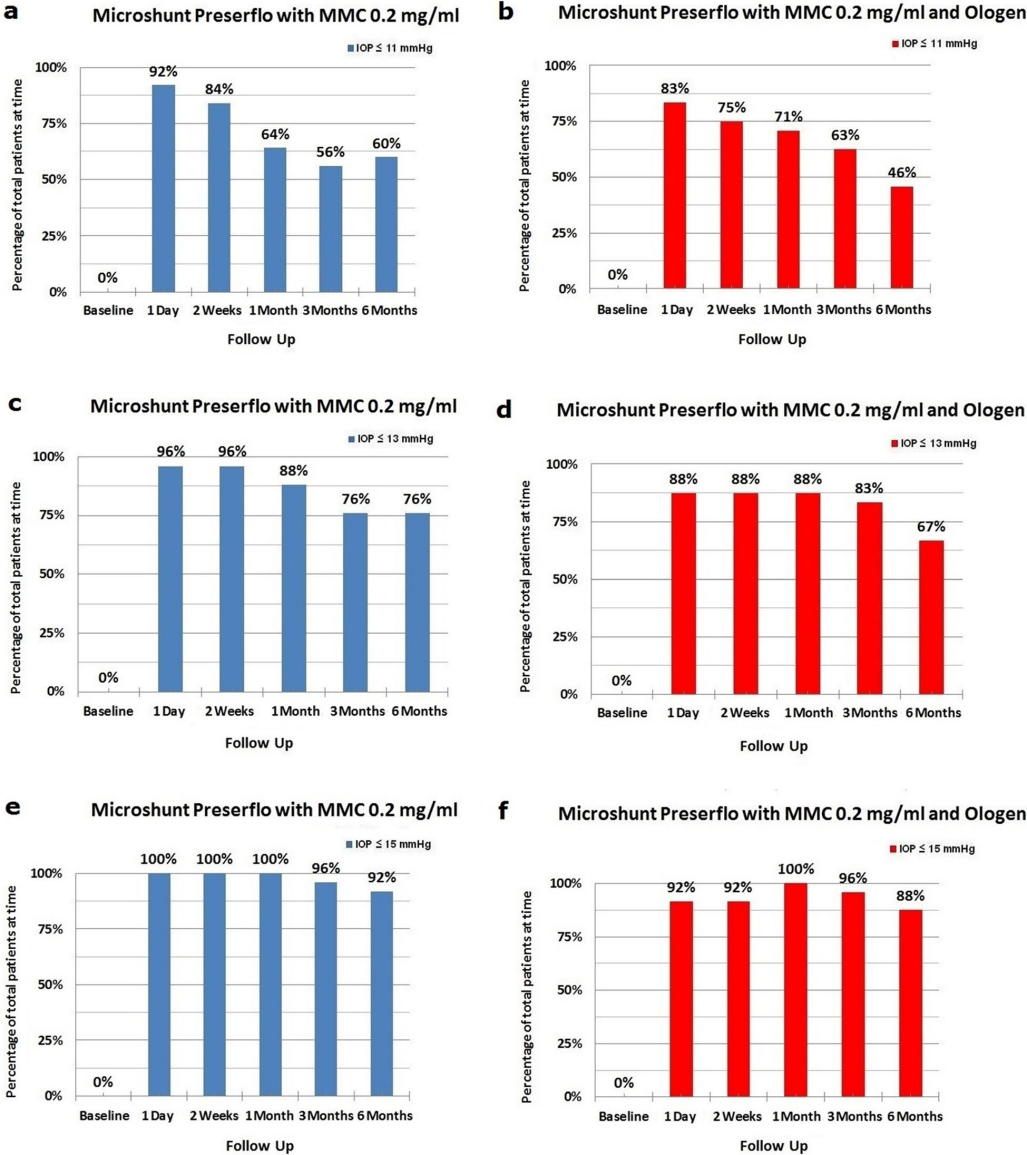
Cumulative intraocular pressure (IOP) results per group (Preserflo Microshunt with MMC 0.2 mg/ml and Preserflo Microshunt with MMC 0.2 mg/ml and Ologen implantation) in percentage for IOP readings less than or equal to (a, b) 11 mmHg, (c, d) 13 mmHg and (e, f) 15 mmHg

Thirty five cases were classified as advanced POAG (with a mean visual field mean deviation (MD) − 20.50 ± 6.38 dB) and 15 cases as moderate (mean visual field MD − 7.37 ± 0.71 dB) according to the Hodapp-Parrish-Anderson (HPA) classification system [23]. Demographics are presented in Table 2. All variables tested for the results presented between both groups A and B were comparable (Table 2). The mean postoperative IOP decreased significantly from baseline in both surgical groups. IOP in group A decreased from 23.52 ± 5.78 to 11.56 ± 3.08 mmHg (49.06% mean IOP reduction) and from 26.04 ± 8.76 to 11.75 ± 3.37 mmHg (53.01% mean IOP reduction) in group B by the 6th month (two-way ANOVA, *P* < 0.001; Fig. 1). Medication decreased in both groups compared to baseline (Table 3). Medication use in group A decreased from 2.52 ± 0.91 to 0.04 ± 0.20 (98.02% reduction, a median reduction of 2.5 medications). Medication use in group B decreased from 2.58 ± 0.82 to 0.16 ± 0.81 (94.44% reduction, a median reduction of 2.5 medications) by the 6th month (Wilcoxon test, *P* < 0.001). The Levene’s equality test was statistically significant (*P* < 0.001, Table 4), and thus the data tested failed to meet the assumption of homogeneity and outliers assumption between the groups. Regarding the power of the samples, we found that the follow-up was the only statistically significant factor (*P* < 0.001) in terms of IOP reduction with partial eta squared 0.63 (63%) and observed power of 1. No other factor or pairwise factor was significant in terms of IOP reduction (Table 4). CDVA was stable in both groups (paired samples t-test, *P* = 0.95, from baseline 0.64 ± 0.24 to the 6th month 0.66 ± 0.21 Snellen and *P* = 0.33, from baseline 0.58 ± 0.27 to the 6th month 0.61 ± 1.93 Snellen, in groups A and B, respectively). Absolute success rates in group A accounted for a) 48%, b) 64% and c) 68% in comparison to group B a) 45.8%, b) 45.8% and c) 58.3% at 6 months (Figs. 2, 3 and 4). Qualified success rate in group A accounted for a) 68%, b) 88% and c) 92% in comparison to group B a) 70.8%, b) 91.8% and c) 95.8% at 6 months (Figs. 5, 6 and 7). Log-rank Kaplan-Meier showed no statistical differences between the groups and parameters tested (*P* < 0.68, *P* < 0.22, *P* < 0.48 absolute success and *P* = 0.38, *P* < 0.31 and *P* < 0.58 qualified success, group A and group B, respectively). The scatterplot analysis verified those results (Fig. 8) by showing a 20% IOP reduction and an overall IOP reduction per case for all 50 eyes in both groups. The cumulative IOP results per group were not statistically different in group A compared to group B at 6 months (Fig. 9 and Table 4). We did not observe any severe intraoperative complications in either group. In the early postoperative follow-up, 12 eyes (24%) presented with transient hypotony (IOP < 5 mmHg) for 1 week, in which 6 of them (50%) presented a minor choroidal detachment in the retinal periphery which resolved within 14 days, while in 1 eye (2%) the choroidal detachment persisted for over 1 month and spontaneously resolved after immediate discontinuation of the corticosteroid topical treatment. Twenty-six eyes (52%) had a normal postoperative follow-up without any interventions or complications. Four revision surgeries due to bleb fibrosis were performed at 6 months and were considered failures (8%). Three revision surgeries were performed at 6 months in group B (12%) in comparison to one in group A (4%). Regarding bleb fibrosis, both groups shared similar characteristics in terms of age, prior surgery status and ethnicity (Table 5). All eyes were conjunctiva surgery naive prior to Preserflo Microshunt implantation and had only undergone cataract extraction. During revision surgeries, the conjunctiva, Tenon’s capsule, sclera, and the Preserflo Microshunt were examined. After removal of fibrosis, the Preserflo Microshunt was checked for flow. If flow was inadequate, the tube was removed from the scleral pocket and flushed with BSS. Upon successful irrigation and adequate flow, the tube was returned back into position following scleral treatment with 0.2 mg/ml MMC for 3 min.

**Table 2:** Demographics of study participants

Demographics	n		
No. of patients	50		
No. of female patients	33		
Age (years)	76.13 ± 10.08		
No. of male patients	17		
Age (years)	79.00 ± 7.94		
No. of eyes operated total (POAG refractory to medical treatment)	50		
Baseline MD (dB) total	−13.31 ± 7.93		
Advanced POAG, Baseline MD (dB) (n = 35)	−20.50 ± 6.38		
Moderate POAG, Baseline MD (dB) (n = 15)	−7.37 ± 0.71		
	**Group A** (n)	**Group B** (n)	**Intergroup variable comparison** t-test, *P* < 0.05 (95% CI)
No. of eyes operated total	25	25	N.A
No. of pseudophakic eyes	25	25	N.A
Age (years)	77.58 ± 7.62	75.64 ± 12.61	0.88 (−6.15 to 5.32)
Baseline MD (dB)	−13.15 ± 8.53	−13.47 ± 7.44	0.84 (−4.57 to 3.78)
Baseline average RNFL thickness (μm)	65.04 ± 8.03	64.54 ± 10.67	0.52 (−3.63 to 6.78)
Baseline IOP (mmHg)	23.52 ± 5.78	26.04 ± 8.76	0.26 (−1.93 to 6.77)
Baseline antiglaucoma agents	2.52 ± 0.91	2.58 ± 0.82	0.36 (−0.38 to 0.98)
Baseline DCVA (Snellen)	0.64 ± 0.24	0.58 ± 0.27	0.43 (−0.23 to 0.10)

*POAG* primary open-angle glaucoma; *IOP* intraocular pressure; *MD* mean deviation; *dB* decibels; *RNF* retinal nerve fiber layer; *CDVA* corrected distance visual acuity; *MMC* mitomycin C; *N.A* not available

Group A: Preserflo Microshunt with MMC 0.2 mg/ml

Group B: Preserflo Microshunt with MMC 0.2 mg/ml and Ologen

**Table 3:** Paired samples t-test comparison of the means in terms of medication in both groups

Follow Up	Intervention	n	Mean ± SD	Std. Error	95% Confidence interval	*P*
Baseline	Group A	25	2.52 ± 0.91	0.19	2.10 to 2.89	0.70
	Group B	25	2.58 ± 0.82	0.16	2.23 to 2.93	
1 Day	Group A	25	0.00 ± 0.00	0.00	0.00 to 0.00	0.00
	Group B	25	0.00 ± 0.00	0.00	0.00 to 0.00	
2 Weeks	Group A	25	0.00 ± 0.00	0.00	0.00 to 0.00	0.00
	Group B	25	0.00 ± 0.00	0.00	0.00 to 0.00	
1 Month	Group A	25	0.00 ± 0.00	0.00	0.00 to 0.00	0.00
	Group B	25	0.00 ± 0.00	0.00	0.00 to 0.00	
3 Months	Group A	25	0.00 ± 0.00	0.00	0.00 to 0.00	0.00
	Group B	25	0.00 ± 0.00	0.00	0.00 to 0.00	
6 Months	Group A	25	0.04 ± 0.20	0.04	−0.04 to 0.12	0.47
	Group B	25	0.16 ± 0.66	0.16	−0.17 to 0.51	

Group A: Preserflo Microshunt with MMC 0.2 mg/ml

Group B: Preserflo Microshunt with MMC 0.2 mg/ml and Ologen

**Table 4:** Two-way ANOVA with tests of between-subjects effects for all variables tested, estimated marginal means and pairwise comparison for the IOP dependent variable and intergroup t-test comparison between Preserflo Microshunt with MMC 0.2 mg/ml and Preserflo Microshunt with MMC 0.2 mg/ml and Ologen implantation

Two-way ANOVA							
**Levene’s test for equality of error variances**							
**F**	**DF 1**		**DF 2**		***P***		
3.9319	11		271		< 0.001		
**Tests of between-subjects effects (all variables)**							
**Source**	**Sum of squares**	**DF**	**Mean square**	**F**	**Partial eta squared**	**Observed power**^**b**^	***P***
Follow up	9514.47	5	1902.89	98.70	0.639	1.0	< 0.001
Intervention	36.31	1	36.31	1.88	0.06	0.27	0.17
Follow up*Intervention	64.22	5	12.84	0.66	0.01	0.25	0.64
Residual	5224.34	271	19.27				
**Estimated marginal means (IOP dependent)**							
**Follow up**		**n**	**Mean**		**Std. Error**	**95% Confidence interval**	
Baseline		50	24.78		0.62	23.54 to 26.01	
1 Day		50	7.37		0.62	6.13 to 8.60	
2 Weeks		50	9.48		0.62	8.24 to 10.71	
1 Month		50	10.12		0.62	8.89 to 11.36	
3 Months		50	10.98		0.62	9.74 to 12.21	
6 Months		50	12.18		0.71	10.78 to 13.58	
**Follow up**	**Intervention**	**n**	**Mean ± SD**		**Std. Error**	**t-test (*P*)**	**95% Confidence interval**
Baseline	Group A	25	23.52 ± 5.88		0.87	0.26	21.79 to 25.24
	Group B	25	26.04 ± 8.76		0.89		24.27 to 27.80
1 Day	Group A	25	7.24 ± 3.47		0.87	0.76	5.51 to 8.96
	Group B	25	7.50 ± 4.23		0.89		5.73 to 9.26
2 Weeks	Group A	25	8.88 ± 3.08		0.87	0.34	7.15 to 10.60
	Group B	25	10.08 ± 5.48		0.89		8.31 to 11.84
1 Month	Group A	25	9.92 ± 3.25		0.87	0.49	8.19 to 11.64
	Group B	25	10.33 ± 2.53		0.89		8.56 to 12.09
3 Months	Group A	25	11.20 ± 2.71		0.89	0.63	9.44 to 12.97
	Group B	25	10.76 ± 2.78		0.87		9.03 to 12.48
6 Months	Group A	25	12.00 ± 3.13		0.63	0.89	10.01 to 13.98
	Group B	25	12.36 ± 3.37		0.67		10.38 to 14.35
**Pairwise comparisons (IOP dependent)**			**Mean difference**		**Std. Error**	***P***^**a**^	**95% CI**^**a**^
Baseline		1 Day	17.41		0.88	< 0.0001	14.78 to 20.03
		2 Weeks	15.29		0.88	< 0.0001	12.67 to 17.92
		1 Month	14.65		0.88	< 0.0001	12.02 to 17.28
		3 Months	13.79		0.88	< 0.0001	11.16 to 16.42
		6 Months	12.59		0.94	< 0.0001	9.78 to 15.40
1 Day		Baseline	−17.41		0.88	< 0.0001	−20.03 to −14.78
		2 Weeks	−2.11		0.88	0.27	−4.73 to 0.51
		1 Month	−2.75		0.88	0.0314	−5.38 to −0.12
		3 Months	−3.61		0.88	0.0009	−6.24 to −0.98
		6 Months	−4.81		0.94	< 0.0001	−7.62 to −2.00
**Pairwise comparisons (IOP dependent)**			**Mean difference**		**Std. Error**	***P***^**a**^	**95% CI**^**a**^
2 Weeks		Baseline	−15.29		0.88	< 0.0001	− 17.92 to −12.67
		1 Day	2.11		0.88	0.27	− 0.51 to 4.73
		1 Month	−0.64		0.88	1.00	−3.27 to 1.98
		3 Months	−1.50		0.88	1.00	−4.13 to 1.12
		6 Months	−2.70		0.94	0.07	−5.51 to 0.10
1 Month		Baseline	−14.65		0.88	< 0.0001	−17.28 to −12.02
		1 Day	2.75		0.88	0.0314	0.12 to 5.38
		2 Weeks	0.64		0.88	1.00	−1.98 to 3.27
		3 Months	−0.85		0.88	1.00	−3.48 to 1.77
		6 Months	−2.05		0.94	0.46	−4.86 to 0.75
3 Months		Baseline	−13.79		0.88	< 0.0001	−16.42 to −11.16
		1 Day	3.61		0.88	0.0009	0.98 to 6.24
		2 Weeks	1.50		0.88	1.00	−1.12 to 4.13
		1 Month	0.85		0.88	1.00	−1.77 to 3.48
		6 Months	−1.20		0.94	1.00	−4.01 to 1.61
6 Months		Baseline	−12.59		0.94	< 0.0001	−15.40 to −9.78
		1 Day	4.81		0.94	< 0.0001	2.00 to 7.62
		2 Week	2.70		0.94	0.07	−0.10 to 5.51
		1 Month	2.05		0.94	0.46	−0.75 to 4.86
		3 Months	1.20		0.94	1.00	−1.61 to 4.01

^a^ Bonferroni corrected

^b^ Computed using alpha = 0.05

Group A: Preserflo Microshunt with MMC 0.2 mg/ml

Group B: Preserflo Microshunt with MMC 0.2 mg/ml and Ologen

SD: standard deviation; DF: degrees of freedom; Eta: eta coefficient (An eta coefficient test is a method for determining the strength of association between a categorical variable)

**Table 5:** Bleb failure risk factors and failure results per group

Demographics	Group A	Group B
No. of eyes operated total	25	25
Prior ocular surgeries	Phacoemulsification	Phacoemulsification
Ethnicity of patients	Caucasian	Caucasian
No. of female patients	18	15
Age (years)	77.58 ± 7.62	75.64 ± 12.61
No. of male patients	7	10
Bleb revision surgery at 6 months (failure rate)	1 (4%)	3 (12%)

*MMC* mitomycin C

Group A: Preserflo Microshunt with MMC 0.2 mg/ml

Group B: Preserflo Microshunt with MMC 0.2 mg/ml and Ologen

## Discussion

Trabeculectomy is still regarded by many surgeons as the gold standard in surgical treatment of POAG patients [15–17, 24]. Nonetheless, its high complication rate and demanding postoperative management have caused surgeons to seek alternative surgical approaches [25]. Such approaches include the XEN Glaucoma Gel Microstent implant (XEN-GGM, Allergan Plc., Parsippany, New Jersey), an ab interno alternative approach to trabeculectomy, which has gained vast popularity in recent years [26, 27]. XEN reduces IOP significantly and is designed to avoid postoperative hypotony which is a major advantage over the traditional trabeculectomy approach. Studies have however shown that in the long term, additional revision surgeries due to bleb fibrosis ranging from 32 to 37.7% are required to maintain a sufficient low IOP without medication [28, 29]. The Preserflo Microshunt is a relatively novel ab externo alternative to trabeculectomy. There are a few known studies to date following Preserflo Microshunt implantation outcomes [12, 30, 31, 32, 33]. Pinchuck et al. [12] first reported on a cohort of 23 successful Preserflo Microshunt implantations in 23 consecutive eyes. In this study, the design and features of the device as well as the steps of the surgical procedure were thoroughly described and reported. The minimal hypotony results in combination with the insignificant bleb inflammation and encapsulation results as well as the effectiveness of the procedure in lowering the IOP postoperatively provided an alternative to primary trabeculectomy. In this study, the IOP was lowered by 50 to 55% reaching a level below 14 mmHg in over 80% of patients. Similar results were reported by Riss et al. in a retrospective two-center, two-surgeon study with one-year follow-up [30]. In that observational study, the primary goal was to demonstrate the significance of different MMC concentrations, 0.4 versus 0.2 mg/ml, and different placement of an MMC sponge in anatomical locations, limbus versus deep in the pocket, in terms of IOP and medication reduction in three distinct groups. They reported a sufficient IOP and medication reduction postoperatively ranging from 38 to 55% and 72 to 85% at 12 months, respectively. The effectiveness in IOP and medication reduction following a Preserflo Microshunt implantation has been well documented in the recent years [30, 31, 32, 33]. In our current study, we found comparable results in terms of mean IOP reduction at 6 months from 23.52 ± 5.78 to 11.56 ± 3.08 mmHg and from 26.04 ± 8.76 to 11.75 ± 3.37 mmHg in groups A and B with a 49.06% mean IOP reduction in group A and 53.01% mean IOP reduction in group B. The mean medication reduction in our study was also similar and comparable to other studies results [12, 30, 31, 32, 33] with medication reduction in group A ranging from 2.52 ± 0.91 to 0.04 ± 0.20 medications (98.02% reduction, median reduction of 2.5 medications) and in group B from 2.58 ± 0.82 to 0.16 ± 0.81 medications (94.44% reduction, median reduction of 2.5 medications) by the 6th month. In terms of absolute and qualified success, different criteria were used in all the studies so an accurate comparison is not possible. Battle et al. [32] defined qualified success as having an IOP of less than 14 mmHg and a minimum IOP reduction of 20% (IOP ≤ 14 mmHg and IOP reduction ≥ 20%). Success rate reported was 100%, 91%, and 95% for the 1st, 2nd and 3rd year, respectively. Schlenker et al. [33] defined absolute success as an IOP greater than 6 and less than 17 mmHg without hypotony (6 < IOP < 17 mmHg) and qualified as the same range with glaucoma medications with at least a 20% IOP reduction. Complete success was achieved in 76.9% of eyes, qualified success in 92.5%. Complete success was 75.6% for an upper IOP cut-off of 14 mmHg and 76.9% for 21 mmHg, and qualified success was 91.9 and 92.5%. Needling rate was 8.5%. Durr et al. similarly to Schlenker et al. [33] defined failure as an IOP less than 6 mmHg with vision loss, greater than 17 mmHg or less of 20% reduction in IOP without medication. Secondary outcomes included thresholds of 6 to 14 mmHg and 6 to 21 mmHg for both complete (no medication) and qualified (with medication) success as well as qualified success for thresholds of 6 to 17 mmHg. A 61.0% complete success and 79.7% qualified success rate was reported. Needling was performed in 11.8% of eyes. We reported comparable results with absolute success rate in group A reaching a) 48%, b) 64% and c) 68% in comparison to group B a) 45.8%, b) 45.8% and c) 58.3% at 6 months (Figs. 2, 3 and 4 respectively). Our qualified success rate in group A was a) 68%, b) 88% and c) 92% in comparison to group B a) 70.8%, b) 91.8% and c) 95.8% at 6 months (Figs. 5, 6 and 7 respectively). Four revision surgeries due to bleb fibrosis were performed at 6 months and were considered failures (8%). Three revision surgeries were performed at 6 months in group B (12%) in comparison to one in group A (4%). In our cohort, a concentration of MMC 0.2 mg/ml was used in all cases with placement of the soaked sponges posteriorly or deep in the pocket for 3 min. We found that the results of Riss et al. in their group C of 38% mean IOP reduction with MMC 0.4 mg/ml scleral treatment deep in the pocket at one-year follow-up were not comparable with our results. In the studies by Schlenker and Durr, [32, 33] different MMC dose of 0.2 versus 0.4–0.5 mg/ml were used in POAG versus secondary open angle glaucoma (SOAG). Our cohort included only POAG eyes with conjunctival naive patients. Other differing factors between the studies of Battle, Durr and Schlenker [31, 32, 33] and ours, were the cohorts’ ethnic group, primarily Afro-Caribbean versus Caucasian, combined surgery with phacoemulsification versus standalone procedure, different MMC dose and the OCM implantation. Regarding the possible benefit provided from OCM implantation we did not find any statistical differences in terms of IOP reduction between the groups at 6 months. In group B, we performed two additional revision surgeries in comparison to group A, although both groups shared almost identical characteristics in terms of bleb failure risk (Table 5). Our current results showed no statistical difference between groups A and B. Similar results were reported in the survival analysis (Figs. 2 and 3) and in the scatterplot analysis (Fig. 8) demonstrating equal IOP reduction among the groups. Our cumulative IOP results also did not demonstrate any statistical differences between groups A and B at 6 months (Fig. 9). In our opinion, an OCM additional implantation could have been of benefit in a cohort with mixed ethnic origin patients other than Caucasian, prior glaucoma surgery and those on long term glaucoma medication.

## Conclusion

We were able to reproduce similar results to pre-existing studies [11, 29, 30, 31, 32, 33]. We did not report any adverse or severe sight-threatening complications and our revision surgery rate was 8%. Our greatest limitation is only having 6 months of data, the retrospective nature of the study and failing the assumption of homogeneity of variance. We advise caution when interpreting our results in terms of OCM evaluation and IOP or medication reduction as well as the current revision rates presented. Long-term follow-up is required to evaluate any benefit in terms of wound modulation of an additional OCM implantation following Preserflo Microshunt surgery.

## Data Availability

Not applicable.

## References

[1] Quigley HA. Number of people with glaucoma worldwide. Br J Ophthalmol. 1996;80(5):389–93. 10.1136/bjo.80.5.389 PMC505485 8695555

[2] Tham YC, Li X, Wong TY, Quigley HA, Aung T, Cheng CY. Global prevalence of glaucoma and projections of glaucoma burden through 2040: a systematic review and meta-analysis. Ophthalmology. 2014;121(11):2081–90. 10.1016/j.ophtha.2014.05.013 24974815

[3] Heijl A, Leske MC, Bengtsson B, Hyman L, Bengtsson B, Hussein M, et al. Reduction of intraocular pressure and glaucoma progression: results from the Early Manifest Glaucoma Trial. Arch Ophthalmol. 2002;120(10):1268–79. 10.1001/archopht.120.10.1268 12365904

[4] Kass MA, Heuer DK, Higginbotham EJ, Johnson CA, Keltner JL, Miller JP, et al. The Ocular Hypertension Treatment Study: a randomized trial determines that topical ocular hypotensive medication delays or prevents the onset of primary open-angle glaucoma. Arch Ophthalmol. 2002;120(6):701–13; discussion 829-30. 10.1001/archopht.120.6.701 12049574

[5] Lichter PR, Musch DC, Gillespie BW, Guire KE, Janz NK, Wren PA, et al. Interim clinical outcomes in the Collaborative Initial Glaucoma Treatment Study comparing initial treatment randomized to medications or surgery. Ophthalmology. 2001;108(11):1943–53. 10.1016/s0161-6420(01)00873-9 11713061

[6] Sawchyn AK, Slabaugh MA. Innovations and adaptations in trabeculectomy. Curr Opin Ophthalmol. 2016a;27(2):158–63. 10.1097/ICU.0000000000000236 26595849

[7] Lavia C, Dallorto L, Maule M, Ceccarelli M, Fea AM. Minimally-invasive glaucoma surgeries (MIGS) for open angle glaucoma: a systematic review and meta-analysis. PLoS One. 2017;12(8):e0183142. 10.1371/journal.pone.0183142 PMC5574616 28850575

[8] King AJ, Shah A, Nikita E, Hu K, Mulvaney CA, Stead R (2018). Subconjunctival draining minimally-invasive glaucoma devices for medically uncontrolled glaucoma. Cochrane Database Syst Rev.

[9] Cutolo CA, Iester M, Bagnis A, Bonzano C, Negri L, Olivari S, et al. Early postoperative intraocular pressure is associated with better pressure control after XEN implantation. J Glaucoma. 2020;29(6):456–60. 10.1097/IJG.0000000000001501 32205829

[10] Mansouri K, Guidotti J, Rao HL, Ouabas a, D'Alessandro E, Roy S, et al. prospective evaluation of standalone XEN gel implant and combined phacoemulsification-XEN gel implant surgery: 1-year results. J Glaucoma. 2018;27(2):140–7. 10.1097/IJG.0000000000000858 29271806

[11] Pinchuk L, Riss I, Batlle JF, Kato YP, Martin JB, Arrieta E, et al. The use of poly (styrene-block-isobutylene-block-styrene) as a microshunt to treat glaucoma. Regen Biomater. 2016;3(2):137–42. 10.1093/rb/rbw005 PMC4817329 27047682

[12] Pinchuk L, Riss I, Batlle JF, Kato YP, Martin JB, Arrieta E, et al. The development of a micro-shunt made from poly (styrene-block-isobutylene-block-styrene) to treat glaucoma. J Biomed Mater Res B Appl Biomater. 2017;105(1):211–21. 10.1002/jbm.b.33525 PMC5215625 26380916

[13] Pinchuk L, Wilson GJ, Barry JJ, Schoephoerster RT, Parel JM, Kennedy JP. Medical applications of poly (styrene-block-isobutylene-block-styrene) (“SIBS”). Biomaterials. 2008;29(4):448–60. 10.1016/j.biomaterials.2007.09.041 17980425

[14] Cillino S, Di Pace F, Cillino G, Casuccio A. Biodegradable collagen matrix implant vs mitomycin-C as an adjuvant in trabeculectomy: a 24-month, randomized clinical trial. Eye (Lond). 2011;25(12):1598–606. 10.1038/eye.2011.219 PMC3234465 21921953

[15] Fili S, Seddig S, Kohlhaas M. Long-term results after trabeculectomy combined with mitomycin C and Ologen implant. Klin Monatsbl Augenheilkd. 2019;236(9):1107–14. 10.1055/s-0044-101461 29590686

[16] Papaconstantinou D, Georgalas I, Karmiris E, Diagourtas A, Koutsandrea C, Ladas I, et al. Trabeculectomy with OloGen versus trabeculectomy for the treatment of glaucoma: a pilot study. Acta Ophthalmol. 2010;88(1):80–5. 10.1111/j.1755-3768.2009.01753.x 19900209

[17] Rho S, Sung Y, Ma KT, Rho SH, Kim CY. Bleb analysis and short-term results of biodegradable collagen matrix-augmented Ahmed glaucoma valve implantation: 6-month follow-up. Invest Ophthalmol Vis Sci. 2015;56(10):5896–903. 10.1167/iovs.15-17480 26348639

[18] Chen X, Yuan F. Ologen implantation versus conjunctival autograft transplantation for treatment of pterygium. J Ophthalmol. 2018;2018:1617520. 10.1155/2018/1617520 PMC6142751 30254754

[19] Picht G, Grehn F. Classification of filtering blebs in trabeculectomy: biomicroscopy and functionality. Curr Opin Ophthalmol. 1998;9(2):2–8. 10.1097/00055735-199804000-00002 10180508

[20] Picht G, Grehn F. Development of the filtering bleb after trabeculectomy. Classification, histopathology, wound healing process. Ophthalmologe. 1998;95(5):W380–7. 10.1007/s003470050285 9643029

[21] Klink J, Schmitz B, Lieb WE, Klink T, Grein HJ, Sold-Darseff J, et al. Filtering bleb function after clear cornea phacoemulsification: a prospective study. Br J Ophthalmol. 2005;89(5):597–601. 10.1136/bjo.2004.041988 PMC1772619 15834092

[22] Hodapp E, Parrish RK, Anderson DR (1993). Clinical decisions in glaucoma.

[23] Sawchyn AK, Slabaugh MA. Innovations and adaptations in trabeculectomy. Curr Opin Ophthalmol. 2016;27(2):158–63. 10.1097/ICU.0000000000000236 26595849

[24] Zahid S, Musch DC, Niziol LM. Lichter PR; Collaborative Initial Glaucoma Treatment Study Group. Risk of endophthalmitis and other long-term complications of trabeculectomy in the Collaborative Initial Glaucoma Treatment Study (CIGTS). Am J Ophthalmol. 2013;155(4):674–80. 10.1016/j.ajo.2012.10.017 PMC3608803 23246272

[25] Lenzhofer M, Kersten-Gomez I, Sheybani A, Gulamhusein H, Strohmaier C, Hohensinn M, et al. Four-year results of a minimally invasive transscleral glaucoma gel stent implantation in a prospective multi-centre study. Clin Exp Ophthalmol. 2019;47(5):581–7. 10.1111/ceo.13463 PMC6767491 30578661

[26] Gillmann K, Bravetti GE, Mermoud A, Rao HL, Mansouri K. XEN Gel Stent in pseudoexfoliative glaucoma: 2-year results of a prospective evaluation. J Glaucoma. 2019;28(8):676–84. 10.1097/IJG.0000000000001295 31162174

[27] Mansouri K, Gillmann K, Rao HL, Guidotti J, Mermoud A. Prospective evaluation of XEN Gel implant in eyes with pseudoexfoliative glaucoma. J Glaucoma. 2018;27(10):869–73. 10.1097/IJG.0000000000001045 30095603

[28] Buffault J, Baudouin C, Labbé A. XEN® Gel Stent for management of chronic open angle glaucoma: a review of the literature. J Fr Ophtalmol. 2019;42(4):391–403. 10.1016/j.jfo.2018.10.005 30879831

[29] Riss I, Batlle J, Pinchuk L, Kato YP, Weber BA, Parel JM. One-year results on the safety and efficacy of the InnFocus MicroShunt™ depending on placement and concentration of mitomycin C. J Fr Ophtalmol. 2015;38(9):855–60. 10.1016/j.jfo.2015.05.005 26363923

[30] Sadruddin O, Pinchuk L, Angeles R, Palmberg P (2019). Ab externo implantation of the MicroShunt, a poly (styrene-block-isobutylene-block-styrene) surgical device for the treatment of primary open-angle glaucoma: a review. Eye Vis (Lond).

[31] Batlle JF, Fantes F, Riss I, Pinchuk L, Alburquerque R, Kato YP, et al. Three-year follow-up of a novel aqueous humor microshunt. J Glaucoma. 2016;25(2):e58–65. 10.1097/IJG.0000000000000368 26766400

[32] Schlenker MB, Durr GM, Michaelov E, Ahmed IIK. Intermediate outcomes of a novel standalone ab externo SIBS microshunt with mitomycin C. Am J Ophthalmol. 2020;215:141–53. 10.1016/j.ajo.2020.02.020 32173344

[33] Durr GM, Schlenker MB, Samet S, Ahmed IIK. One-year outcomes of stand-alone ab externo SIBS microshunt implantation in refractory glaucoma. Br J Ophthalmol. 2020 Oct;23 bjophthalmol-2020-317299. 10.1136/bjophthalmol-2020-317299 33097520

